# Using an online community of practice to explore the informal use of mobile phones by health workers

**DOI:** 10.1093/oodh/oqac003

**Published:** 2023-01-04

**Authors:** Claire Glenton, Josephine Nabukenya, Smisha Agarwal, Michele Meltzer, Elisabeth Mukendi, Immaculate Nakityo Lwanga, Josephine Namitala, Sunanda Reddy, Geoff Royston, Tigest Tamrat, Neil Pakenham-Walsh

**Affiliations:** Department of Health and Functioning, Western Norway University of Applied Sciences, 5020 Bergen, Norway; Department of Information Systems, School of Computing & Informatics Technology, Makerere University, 7062, Kampala, Uganda; Department of International Health, Johns Hopkins University Bloomberg School of Public Health, Baltimore, MD 20205, USA; Department of Medicine, Thomas Jefferson University, Philadelphia, PA 19107, USA; Department of Health, Senghor University, Alexandria Governorate 5361022, Egypt; School of Public Health, Makerere University, 7062, Kampala, Uganda; Department of Adult and Community Education, Makerere University, 7062, Kampala, Uganda; Centre for Applied Research and Education on Neurodevelopmental Impairments and Disability-related Health Initiatives, New Delhi 110075, India; Healthcare Information for All, Global Healthcare Information Network, Oxfordshire, UK; UNDP/UNFPA/UNICEF/World Bank Special Program of Research, Development and Research Training in Human Reproduction (HRP), Department of Sexual and Reproductive Health and Research, World Health Organization, Geneva 1211, Switzerland; Healthcare Information for All, Global Healthcare Information Network, Oxfordshire, UK

**Keywords:** informal mhealth, digital innovation, health workforce, digital health, mobile health, mobile device

## Abstract

Health workers are increasingly harnessing mobile phones to develop their own solutions to work challenges. The mHEALTH-INNOVATE project aims to explore this topic further. In 2022, Healthcare Information for All organized an online discussion among health workers and other stakeholders to inform the project. Twenty-five people joined the discussion. Contributors’ descriptions of the varieties of mobile phone use tallied with previous research, including for communication with patients and colleagues. In addition, they described increased mobile phone use in response to the COVID-19 pandemic and the increased need for communication, monitoring and reporting, including during lockdowns. Some solutions were health worker-initiated, including the establishment of WhatsApp groups. The discussion has helped develop a definition of informal mobile phone use.

## BACKGROUND

In 2019, the World Health Organization (WHO) published its first guidelines on the use of digital health strategies for health systems strengthening [1]. These guidelines were informed by a series of Cochrane Reviews exploring the effectiveness, acceptability and feasibility of mobile phones for a range of tasks, including health worker training and decision-support, stock notification, and telemedicine [2–11]. The reviews highlight a range of challenges when mobile phone-based strategies initiated by researchers, governments and others are implemented, and a need for new ideas about how these challenges can be overcome.

Since the WHO guidelines were published, access to low-cost mobile phones and to the internet has increased further. In addition, the COVID-19 pandemic has placed enormous pressure on health systems. Faced with these circumstances, health workers are increasingly using their mobile phones to develop their own strategies and solve the problems they face in their everyday work [12]. This can include the use of easily accessible messaging apps, such as WhatsApp, to communicate and provide support to one another [13]. It can also include informal approaches to gathering, storing and sharing information including clinical or logistical data. These solutions could represent innovative strategies that formal health systems can learn from, but they could also create challenges of their own. Either way, they warrant further exploration.

‘mHEALTH-INNOVATE’ is a 4-year research project (2022–2025) (https://www.fhi.no/en/cristin-projects/ongoing/mhealth-innovate/) funded by the Norwegian Research Council. The project will study informal mobile phone practices among health workers through a systematic review of the global literature as well as through primary research in Uganda. It will also assess whether these informal solutions can be integrated into health policies and governance to enhance service delivery and will lead to a co-produced evidence brief for policy. However, one of the challenges faced by the project is the fact that informal mobile phone use among health workers is still a relatively unexplored topic. This means that we lack a good understanding, as well as a clear definition or classification of the phenomenon we wish to explore.

To help inform further development of the project, Healthcare Information for All (HIFA) organized an online discussion on health workers’ informal use of mobile phones in April/May 2022 (https://www.hifa.org/projects/new-mhealth-innovate-what-can-we-learn-health-workers-informal-use-mobile-phones). HIFA is a global health community of practice, working towards the reduction of avoidable death and suffering by improving the availability of reliable healthcare information and protecting people from harmful misinformation. It has 20 000 professional members from 180 countries representing frontline health workers, patients, general public, policymakers, researchers, journal publishers, systematic reviewers, guideline developers, producers of reference and learning materials, journalists, social scientists, library and information professionals.

The aim of the HIFA discussion was to help the mHEALTH-INNOVATE project explore ways in which health workers are currently using mobile phones, the circumstances that have prompted this use and any self-initiated solutions that have emerged. We also aimed to use the results of the discussion to help develop a ‘working definition’ of informal mobile phone use.

## METHODS

HIFA established a working group drawn from members of the mHEALTH-INNOVATE research project team and from existing HIFA members with a particular interest in or experience of digital health. The working group discussed and agreed upon five questions to help structure the HIFA discussion.

1. Do you use a mobile phone to help you in your work? How do you use it?
2. How do you use your phone to communicate with colleagues, patients and families?
3. How do you use your phone for other work-related purposes? (For example, searching for healthcare information and guidance, tracking supplies, referring patients...)
4. Can you give any examples of ‘informal’ use of mobile phones by health workers? (That is, uses of mobile phones that have not been directed by the employer, but have been initiated, adapted or developed by health workers themselves.)
5. How can the informal use of mobile phones be improved to strengthen healthcare?

In the lead up to the discussion, the working group publicized the discussion to relevant contacts and networks and invited people to join HIFA in readiness. The questions were then posted on HIFA to stimulate discussion. During the discussion, members of the working group encouraged people to respond to the questions, monitored contributions and asked follow-up questions.

Once the discussion was over, one member (CG) of the working group carried out a thematic analysis of the contributions. She compiled, read and familiarized herself with the contributions, coded and categorized the contents of each contribution and selected and presented illustrative verbatim quotes. Finally, she shared preliminary results with the other members of the working group before further refining the categories.

## RESULTS

Twenty-five people, including eight members of the working group, made a total of 71 contributions to the discussion list. Contributors were based in UK (5), Uganda (3), India (3), USA (2), Switzerland (2), Zimbabwe (1), Cameroon (1), Nigeria (1), Ethiopia (1), Democratic Republic of Congo (1), Haiti (1) Iraq (1), Norway (1), Netherlands (1) and Philippines (1). A total of 19 of the 71 contributions were invitations and follow-up questions from working group members, leaving 62 contributions to analyse.

### Identifying existing literature

Nineteen of the contributions included references to relevant articles and reports (e.g. [14–16]). As the field of informal mobile phone use is still a new topic of research, there is also no consensus around the terms that are used to describe it. It was therefore particularly helpful to receive these suggestions, and all were retrieved and examined for relevance to the working definition of informal use.

### Categorizing contributions

Our thematic analysis of the remaining contributions led us to develop the following categories.

- How are health workers using mobile phones in their work?
- Why are health workers turning to mobile phone-based solutions?
- To which extent are these mobile phone uses informal?
- What challenges and possible solutions arise when health workers use mobile phones?

#### How are health workers using mobile phones in their work?

Contributors described the use of mobile phones to carry out a range of activities, although the extent to which these uses were part of a formal, standardized programme or not was not always clear. These uses included using the phone’s clock, stopwatch, torch, camera and calendar functions, and using the phone to search for information on internet-based resources. However, most contributors described the use of mobile phones to communicate with others.

Contributors described using mobile phones to communicate via phone calls; text messages; social media platforms such as Facebook; and communication apps such as WhatsApp, Viber, Teams, email, Messenger and Skype, with WhatsApp being the most commonly referred to.

Health workers communicated via mobile phone with other health workers or staff members, both in their own facilities and in other parts of the health care system. This included sharing practical, clinical and scientific information and resources with colleagues and offering each other emotional support. It also involved connecting health workers at different levels of the health system; for instance, when facilitating referrals, including the transmission of patient information and organizing transport. Other uses included tracking and requesting drugs and supplies, tracking and registering patient information and submitting reports and statistics.

Health workers also used mobile phones to communicate with patients, patient groups and their families. This included sharing general health information and education and sharing individualized information such as appointment reminders, sending test results, and giving teleconsultations.

Finally, health workers used mobile phones to participate in online training, education, supervision and monitoring of drug supplies.

#### Why are health workers turning to mobile phone-based solutions?

Several contributors described how mobile phones eased their workflow by allowing them to gather, search for and share information quickly. For instance, one UK-based GP described how she leaves her personal mobile phone number when asking specialists to contact her about a clinical concern as she experiences formal communication channels as too slow.

Many contributors described how the COVID-19 pandemic increased the need for mobile phone-based strategies. Reasons for this included the following.

An increased need for information to health workers and patients: contributors described how the pandemic led to a need to spread information quickly and to debunk misinformation, both among health workers and patients:

‘2020 saw us do much Covid education for staff and parents together over phone (dos and don’ts) and reach out to allay the fears and anxiety of children and families during the lockdown.’ (Paediatrician, India)

An increased need for monitoring and reporting of stock levels: one contributor described how the increased need for equipment during the pandemic also led to a need for more frequent tracking and notification of stock levels.

‘During the peak of the pandemic, it was critical to track availability of [personal protective equipment] at health facilities weekly, but the traditional stock tracking system used a monthly and quarterly system. ln order to ensure timeliness of reporting, google forms were created and those with smart phones used these to send weekly updates on stock status.’ (Pharmacist, Zimbabwe)

Decreased facility-based service provision and limited mobility of health workers and patients: the pandemic limited the ability of healthcare facilities to deliver regular services and led to widespread restrictions on the movements of both health workers and patients, particularly during periods of lockdown. Contributors described how these circumstances were partly addressed through mobile phone-based communication.

‘During the COVID-19 lockdowns, health facilities set up informal WhatsApp groups (in Northern Uganda) to ensure quality service delivery. Such groups had the participation of facility health workers, community health workers (Village Health Teams) and local leaders. The facility health workers would use such channels to conduct mentorship sessions after trainings during the lockdowns. Community health workers would also use the same channels to secure first line help, report reactions to medication (Family planning projects) and share weekly progress reports.’ (Project manager, Uganda)

‘Mobile phone during the challenging lonely periods was sometimes the only device for health care workers to connect with each other both for physical and psychological support.’ (Global health professional, Switzerland)

Mobility restrictions outside of pandemics: contributors also referred to the use of mobile phones where mobility was restricted for other reasons than the COVID-19 pandemic. One contributor referred to the usefulness of mobile phones for health workers working in remote areas. Another contributor described the importance of mobile phones in a conflict-affected setting. Here, the impact of the conflict on people’s mobility meant that supervision and reporting normally done by higher-level staff was now left to lower-level staff with the help of mobile phones.

‘In Cameroon where there is a conflict, mobile phones are used to send reports and statistics to the regional level. Health supervisors can no longer move to local health units or District Health Services to perform their duties. Health officials at the local level have become supervisory authorities. Without any formal knowledge to perform these tasks, the local health personnel are required to do this. Even the District Medical Officers who have abandoned their services depend now only on the local health staff for reports. This is done only with the help of a mobile phone. This is an important topic that should be encouraged especially in conflict and disaster zones of the world.’ (Development professional, Cameroon)

#### To which extent is health worker mobile phone use informal?

While one contributor mentioned that most of his mobile phone use ‘is not directed by the institution, and therefore “informal”’, most contributors did not refer specifically to or distinguish between ‘formal’ or ‘informal’ use of mobile phones. Nevertheless, several of the contributions suggest that health workers are circumventing formal systems and establishing their own in order to deliver the tasks expected of them in a timely manner. This includes the UK-based general practitioner using her personal phone number when contacting specialists. It also includes the WhatsApp groups initiated and established by the health workers themselves, for instance the Ugandan health workers’ use of private WhatsApp groups to mentor each other during lockdowns, secure help, report medication reactions and share progress reports. One contributor explained that while the initiative to establish WhatsApp groups came from the health workers, their leadership eventually started encouraging use of these groups as a channel of communication. Another contributor suggested that the COVID-19 pandemic was an emergency situation that helped ‘formalize’ the use of mobile phones.

#### What are the challenges and possible solutions when health workers use mobile phones, both formally and informally?

While contributors were generally positive to the use of mobile phones and the opportunities these offered, they also highlighted several challenges. Several of these challenges have previously been identified in the reviews prepared for the WHO digital health guidelines [1–5], and in other reviews [17–19], and are likely to be seen in both formal and informal strategies. For instance, contributors referred to poor internet access; poor quality online information; a lack of digital platforms in local languages; and health workers having to pay for phones, maintenance and airtime. Similarly, they described how a lack of mobile phones, particularly among marginalized populations, limited health workers’ ability to communicate digitally with their patients. Contributors also pointed to challenges tied to health workers’ own low literacy skills and digital literacy skills. These included health workers not understanding how to use available applications and health workers struggling to type, read or write.

One contributor described how a lack of mobile phones in the community had been partly addressed during the COVID-19 pandemic by pairing families with community health workers who owned mobile phones. Other contributors described how health workers tried to overcome literacy problems by using audio or video recordings to capture information, although this brings with it its own considerations regarding data management.

‘Some [community health workers] resort to audios and video audios and video recordings – recording themselves while describing the client’s condition or situation. They did not record the clients themselves except in situations where it was necessary, and then only recording the specific body part. But this creates a challenge for data management.’ (Project manager, Uganda)

Other challenges described by contributors may be particularly relevant for informal digital strategies. Some contributors were concerned about confidentiality issues, particularly when sharing information about patients. Solutions suggested by contributors included limiting what they discuss on the phone, locking phones and subscribing to antivirus and anti-hacking tools, anonymizing patient data and creating awareness.

‘...because we are told that WhatsApp messages are encrypted, I have used it on occasions to refer patients to known medical colleagues, especially when out of the office. Even then, there are certain details that I leave out for direct communication to the colleagues (there is still the fear/uncertainty about securing confidentiality). I also ensure that my phone has a code (PIN) that allows me open it for use, and it has anti-virus cover to prevent hacking!!’ (Public health professional, Nigeria)

One contributor asked for clarification regarding liability and the legal status of communication that takes place in work-related WhatsApp groups.

‘Do we consider discussions done and resolutions passed on WhatApp platforms as binding eg work assignments given to employees via this platform-would an employee who does not follow through be liable for disciplinary measures? How do we handle harassment issues that originate from discussions on some WhatsApp groups? How do we handle grievances aired on the WhatsApp platforms? Who follows them up?’ (Pharmacist, Zimbabwe)

Contributors called for formal policies on the use of mobile phones when transmitting information. They also called for more guidance regarding the type of information that should or should not be shared and guidance about how to share patient information while anonymizing it. One contributor linked a lack of policies and guidance to the fact that mobile phones were often the personal property of the health worker and that their use is often not part of the formal health care system. Others pointed to the need for governments to start documenting and reviewing informal and innovative use of mobile phones.

### Current working definition of informal mobile phone use

We have used the results of the HIFA discussion, in combination with published research on the topic and input from experts within our research team and its international advisory group, to develop a working definition of health workers’ informal mobile phone use. We define informal mobile phone use as healthcare workers’ use of mobile phones to support their work, using approaches that are initiated by the healthcare workers themselves and that are initially not standardized, regulated or endorsed by the health system or organization to which they belong.

## CONCLUSION

The HIFA discussion only included a small number of participants. However, it enabled a wide range of relevant stakeholders to explore the issues interactively. Contributors described a range of mobile phone uses, reflecting existing descriptions of mHealth use [20]. In particular, health workers used the phone as a communication channel, either with colleagues and other parts of the health system or with patients and families. Contributors also described a range of challenges tied to mobile phone use also identified in previous research of mobile phone use among health workers [3].

What is novel in the HIFA discussion are the many examples of the increased use of mobile phones due to the COVID-19 pandemic. The pandemic appears to have stimulated health care workers to generate informal approaches in their use of mobile phones. This includes the establishment of private WhatsApp groups, often initiated by the health workers themselves to meet the increased needs for information, monitoring and reporting. Challenges with informal solutions highlighted by contributors included confidentiality and liability issues.

In general, contributors did not make a clear distinction between ‘formal’ and ‘informal’ use of mobile phones. However, they did describe some self-initiated mobile phone-based strategies. They also gave at least one example of how health worker-initiated communication channels were gradually encouraged by the leadership, exemplifying how use may transition over time from informal to more formal. More work is needed to crystallize definitional and taxonomic issues about informal use of mobile phones. We also need to explore the practical implications, advantages and disadvantages of informal use, including how this may vary from setting to setting. The involvement of stakeholders through this HIFA discussion is the first step in our exploration of informal digital health and has contributed to our current working definition of informal mobile phone use. We are currently using this definition to scope a systematic review on this topic and to shape the primary research planned as part of the mHEALTH-INNOVATE project.

## Disclosure statement

The HIFA discussion list took place to support the mHEALTH-INNOVATE project that is funded through a research grant from the Research Council of Norway (https://prosjektbanken.forskningsradet.no/en/project/FORISS/325476). The authors declare no financial conflicts of interest.

## Authors’ contributions

N.P.W. conceptualized the HIFA-mHealth discussion and led the development of the discussion, with support from all other co-authors. C.G. carried out the initial data analysis and developed the first draft of the paper. All authors provided comments to and approved this paper.

## Data availability statement

All data from the HIFA discussion is available at the HIFA website: https://www.hifa.org/rss-feeds/17.
